# Fe-Loaded MOF-545(Fe): Peroxidase-Like Activity for Dye Degradation Dyes and High Adsorption for the Removal of Dyes from Wastewater

**DOI:** 10.3390/molecules25010168

**Published:** 2019-12-31

**Authors:** Chuang Zhang, Haichao Li, Chen Li, Zhengqiang Li

**Affiliations:** 1 Key Laboratory for Molecular Enzymology and Engineering of the Ministry of Education, College of Life Sciences, Jilin University, Changchun 130012, China; zhangchuang19@mails.jlu.edu.cn (C.Z.); lihc201234@163.com (H.L.); 2 Key Laboratory for Zoonosis Research, Ministry of Education, Institute of Zoonosis, Jilin University, Changchun 130012, China

**Keywords:** Fe-loaded MOF-545(Fe), peroxidase-like activity, dye absorption, dye degradation, wastewater

## Abstract

Methods to remove dye pollutants with natural enzyme, like horseradish peroxidase (HRP), are still limited due to high costs and low stability levels. The development of such a method with similar enzymatic activity is important and could be helpful in wastewater disposal. A metal organic framework material, Fe-loaded MOF-545 (Fe), was synthesized in our study as a new way to remove dyes due to its peroxidase-like activity. The structural characterizations of Fe-loaded MOF-545(Fe) was investigated using scanning electron microscopy (SEM), UV-Vis absorption spectra, and X-ray diffraction (XRD). The peroxidase-like (POD-like) activity of Fe-loaded MOF-545(Fe) was investigated under different pH and temperature conditions. Because of the Fe added into the MOF-545 structure, the absorption of Fe-loaded MOF-545(Fe) for acid (anionic) dyes (methyl orange (MO)) was better than for basic (cationic) dyes (methylene blue (MB)). The Fe-loaded MOF-545(Fe) could give a significant color fading for MO and MB over a short time (about two hours) with peroxidase-like activity. The remarkable capacity of Fe-loaded MOF-545(Fe) to remove the MO or MB is due to not only physical adsorption, but also degradation of the MO and MB with POD-like activity. Therefore, Fe-loaded MOF-545(Fe) has significant potential regarding dye removal from wastewater.

## 1. Introduction

In recent years, dyes have been widely used to color the products in many industries, such as dyestuffs, paper, leather, plastic, rubber, and cosmetics [1]. Some of the commercial dyes are discharged into the environment directly, causing environmental pollution [2]. Every year, more than 100,000 tons of commercial dyes are consumed in the textile industry, and approximately 100 tons are discarded directly into the water stream [3,4,5,6]. Since most of the dyes are highly toxic, synthetic sources with complex chemical structure, they are stable and biodegradation by the environment is a difficult process [7]. However, colored water with high dyes adsorption gives a bad impression and is considered to be unhealthy for human consumption. Thus, removing dyes from the industrial waste is an important scientific challenge. At present, various methods, such as adsorption, coagulation, advanced oxidation, and membrane separation, are used to eliminate the dye contamination from the industrial waste [8,9,10,11]. Among various techniques, adsorption is considered to be a promising method because of its simplicity and efficiency [5]. Several adsorbents, such as activated carbons, clay materials, and multifunctional nanoparticles are used to remove dyes to purify polluted water [10,11,12,13]. However, most of the mentioned absorbents have pore diameter of less than 2 nm, thereby limiting the capture of bulky dye molecules to the inner surface of the pore. Therefore, a new adsorbent to remove accessible bulky dyes from wastewater is required. Although physical methods of removing dyes from wastewater are helpful, biological methods, which involve catalyzing and degrading the dye molecules via enzymatic properties, have an advantage regarding environmental protection including low sludge and no toxic production. Horseradish peroxidase (HRP) is one of the most common natural enzymes used to eliminate phenols from water solutions, but treatment of large volumes of wastewaters using HRP is difficult due to the high cost [14]. Compared with the limitations of the natural enzyme [13], this specific enzyme (or contain the enzymatic properties) has the stable chemical properties and catalytic abilities, making it possibly suitable to remove dyes from industrial waste. A number of reports described that some natural enzyme activity can be manifested in a new crystalline porous material with a metal-organic frameworks (MOFs).

Metal-organic frameworks (MOFs) are a recent classification of porous material, which are attracting interest due to its applications, such as membrane separation, sensing, catalysis, and proton conduction [15,16,17,18]. The adsorption activity of MOF was affected by the modification of MOF structure. MOF modified by functional group (such as quaternary N-alkyl ammonium salt) and MOF treated by metalation with Cu or Zn all exhibit better adsorption capacities in organic dyes [19,20,21]. Yaghi et al. synthesized a zirconium–metalloporphyrin meso MOF, named MOF-545 [22]. Zhou et al. also reported a similar structure and named it PCN-222, independently [23]. Zr-based MOF-545 was composed of Zr_6_ clusters and TCPPs (tetrakis(4-carboxyphenyl)porphyrin) that supported an open channel with a large diameter (3.7 nm). The metal iron (Mn, Fe, Co, Ni, Cu, Zn, Pd) could be immobilized by a porphyrin frame depending on the application. Because of the large pore size and Zeta potentials for Zr-based MOF-545, our laboratory clarified that it possessed the high absorption ability for dyes in aqueous solutions. Specifically, the adsorption capacity with the same dye enhancement was observed from our precious work [24]. 

From the published results, peroxidases were discovered to be enzymes that catalyzed the degradation of dyes alongside H_2_O_2_ [25]. During the catalytic oxidization for industrial dyes, the peroxidases were extremely stable, while also being cost-effective and with short treatments [26]. Moreover, peroxidases catalyze a wide range of aromatic substrates which open the complex aromatic rings to degrade heterocyclic structures. MOF-based catalysts for photo-degradation of organic dyes have already been published in several reports. Many sorts of MOF complexes exhibited the excellent photocatalytic capability for methyl orange (MO) and methylene blue (MB). The addition of H_2_O_2_, acting as the second ligand, onto the complex enhanced the photo-degradation of organic dyes [27,28]. With the Fe metalized into MOF-545, the Fe-loaded MOF-545(Fe) can catalyze the substrates with its peroxidase-like activity, which avoids the utilization of the complicated photocatalytic systems. The Fe-loaded MOF-545(Fe) was acting as an effective peroxidase mimic with a substrate binding affinity (K_m_) and a catalytic activity (k_cat_) superior to heme in aqueous media. The integration of high-density catalytic centers, ultra-large open channels, and the extraordinary chemical stability of Fe-loaded MOF-545(Fe) suggests a bright future regarding building MOF-based platforms for enzyme-mimicking catalysis [23].

The study of Fe-loaded MOF-545(Fe) clarified that a variety of substrates were catalyzed by Fe-loaded MOF-545(Fe) with excellent binding affinity and catalytic activity [23]. To understanding the character of Fe-loaded MOF-545(Fe) precisely, in this work, the POD-like activity of Fe-loaded MOF-545(Fe) was observed. The Fe-loaded MOF-545(Fe) was characterized using UV-Vis absorption spectra, X-ray diffraction (XRD), scanning electron microscopy (SEM), nitrogen adsorption and, Zeta potential analysis. To clarify the enzymatic properties of Fe-loaded MOF-545(Fe), the classical peroxidase substrate 3,3,5,5-tetra-methyl-benzidine (TMB) was studied in our work. From the published results, the anionic dye MO and cationic dye MB both are widely found in textile wastewater [29,30]. Since the Fe-loaded MOF-545(Fe) was confirmed to exhibit the peroxidase efficacy, the catalytic properties of Fe-loaded MOF-545(Fe) were observed via degradation using methylene blue (MB) and methyl orange (MO). Our results showed that Fe-loaded MOF-545(Fe) was stable and sensitively reactive with substrate, thereby presenting an advantage as a new method in processing wastewater.

## 2. Results and Discussion

### 2.1. Sample Characterization of Fe-Loaded MOF-545(Fe)

#### 2.1.1. Sample Characterization by SEM, XRD, and UV-Vis Absorption Spectra

**Figure 1:**
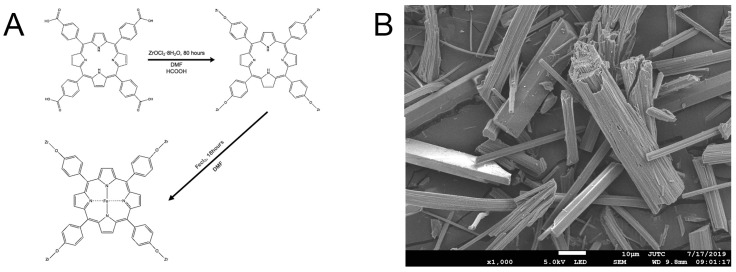
The synthetic graph and the SEM images of Fe-loaded MOF-545(Fe). (**A**) The synthetic process of Fe-loaded MOF-545(Fe), (**B**) the SEM images for Fe-loaded MOF-545(Fe).

**Figure 2:**
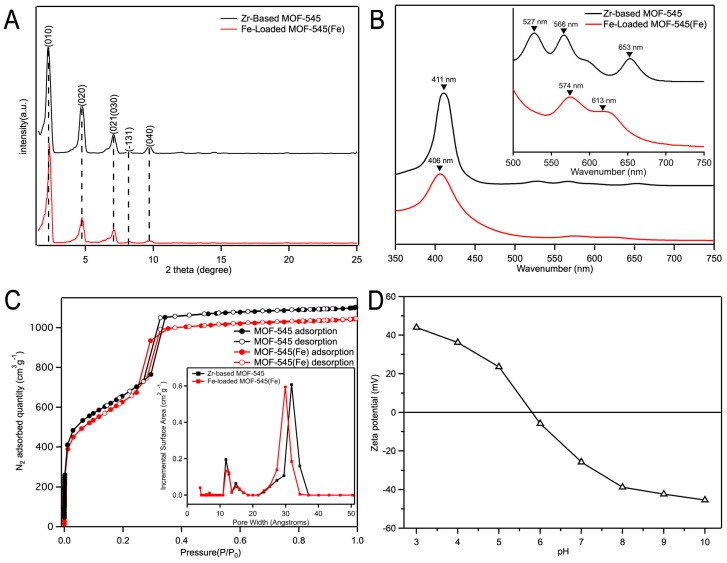
The characterization of Fe-loaded MOF-545(Fe). (**A**) X-ray diffraction (XRD) patterns of Zr-based MOF-545 (black line) and Fe-loaded MOF-545(Fe) (red line), (**B**) the UV-Vis absorption spectra of Zr-based MOF-545 (black line) and Fe-loaded MOF-545(Fe) (red line), (**C**) N_2_ adsorption/desorption isotherms and pore size diameter of the Zr-based MOF-545 and Fe-loaded MOF-545(Fe), (**D**) Zeta potential analysis of Fe-loaded MOF-545(Fe).

The Fe-loaded MOF-545(Fe) was synthesized following the synthetic scheme shown in Figure 1A and the method described in Section 3.1. The structural characterizations of Fe-loaded MOF-545(Fe) were investigated using SEM, XRD, and UV-Vis absorption spectra. The SEM image of Fe-loaded MOF-545(Fe) is shown in Figure 1B. The SEM results showed that the crystal (Fe-loaded MOF-545(Fe)) exhibited a rod-like morphology in size (3.7 nm) with hexagonal edge, which was the same as the published results [6,31]. The introducing of a Fe ion into Zr-based MOF-545 reduced the lattice with the shift of diffraction peaks to high 2-theta degree slightly, as marked by dotted lines in Figure 2A. Similar effect was been found in N_2_ adsorption results, with reduction of adsorption gas volume and decreased pore size (Figure 2C). This effect may result from the polarization effect of Fe^3+^ in the center of porphyrin in Zr-based MOF-545, which caused a synergic effect on the shrinkage of crystal lattice and, therefore, reduction of pore size in Fe-loaded MOF-545(Fe) [23]. The Zr-based MOF-545 and Fe-loaded MOF-545(Fe) were dissolved in 1 M NaOH solution and observed via UV-Vis absorption spectra as shown in Figure 2B, independently. In the Soret band region, the MOF-545 with and without Fe ion exhibited peaks at 406 nm and 414 nm, respectively. The spectra at the Soret band region were related to the vibration model of porphyrin. In the Q-band region of MOF-545, three peaks were detected at 527 nm, 566 nm, and 653 nm separately (Figure 2B inset). Two peaks in the Q-band region of the Fe-loaded MOF-545(Fe) were observed in Figure 2B (inset). The fingerprint nature of the porphyrin species for each MOF was quantitatively metalized, as shown in Figure 2B. The symmetry of porphyrin increased after forming a metal compound in the center, which showed the decreasing peak number in the Q-band region. In our results, the peak position in the Q-band and Soret band regions were consistent with the published results [22], thereby showing that the Fe formed a metal organic framework in the Fe-loaded MOF-545(Fe). The FTIR results of the Fe metalized on the MOF-545 are shown in Figure S1, in which a stretching/bending vibration of the Fe–N bond around 1000 cm^−1^ was observed [32,33,34]. 

#### 2.1.2. N_2_ Adsorption/Desorption Isotherms

The N_2_ adsorption/desorption isotherms of Fe-loaded MOF-545(Fe) and Zr-based MOF-545 were measured to investigate the Barrett–Joyner–Halenda (BJH) pore diameter and the Brunauer–Emmett–Teller (BET) surface area (Figure 2C). The samples exhibited H1-type narrow hysteresis loops, which were classified by the International Union of Pure and Applied Chemistry (IUPAC) [35]. The BJH pore diameter of Fe-loaded MOF-545(Fe) were 1.3 nm and 3.1 nm with BET surface area at 2368 m^2^·g^−1^. The Fe-loaded MOF-545(Fe) showed a slight change in pore size comparing with Zr-based MOF-545. The results were consistent with the XRD described in Figure 2A. Most of the pore size was larger than that of MO and MB (~2 nm), thereby giving an advantage regarding the removal of the dyes [36].

#### 2.1.3. Zeta Potential Analysis of Fe-Loaded MOF-545(Fe)

Zeta potential analysis is an important method, which is related to the adsorption capacity and interaction. Regarding the adsorption capacity of Fe-loaded MOF-545(Fe), the Zeta potential analysis from pH levels of 3.0 to 10.0 is shown in Figure 2D. The pH_PZC_(PZC) of Fe-loaded MOF-545(Fe) was almost close to pH 6.0, whereby the Fe-loaded MOF-545(Fe) existed as electrical neutrally. When the solution was under acidic conditions (pH < 6.0), the H^+^ accumulated on the surface of Fe-loaded MOF-545(Fe), thereby allowing a positive charge to exist. On the other hand, when the solution was under alkaline conditions (pH > 6.0), the OH^−^ group in the solution accumulated on the surface, causing a negative charge Fe-loaded MOF-545(Fe). Because of its electric potential character, the Fe-loaded MOF-545(Fe) was used as the adsorption method in the solution. 

### 2.2. The Enzyme-Like Activities of the Fe-Loaded MOF-545(Fe)

#### 2.2.1. Intrinsic Peroxidase Activity of Fe-Loaded MOF-545(Fe)

**Figure 3:**
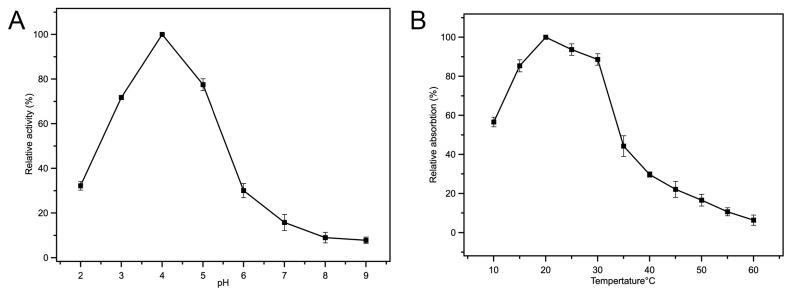
Effect of pH and temperature on Fe-loaded MOF-545(Fe). (**A**) The relative activity of the Fe-loaded MOF-545(Fe) in different pH, (**B**) the peroxidase-like activity of the Fe-loaded MOF-545(Fe) in different temperature.

The peroxidase-like (POD-like) activity of Fe-loaded MOF-545(Fe) was confirmed by tetramethylbenzidine (TMB) as described in the methods section. At 652 nm, TMB had maximum absorbance, which was investigated as a functional mark to test the peroxidase-like activity. As shown in Figure S2, with increasing the reaction time by 5 min, the absorbance of oxidized TMB at 652 nm was increased alongside H_2_O_2_. Based on these results, it was determined that the Fe-loaded MOF-545(Fe) was activated as an intrinsic peroxidase during the TMB reaction. Figure 3A shows the effect of pH on catalytic activity, in the range of pH from 2.0 to 9.0, whereby the catalytic activity of Fe-loaded MOF-545(Fe) was increasing from pH 2.0 to 4.0 and drastically decreased toward inactivity as neutral pH (6.0–8.0) was neared. The most suitable reaction environment for Fe-loaded MOF-545(Fe) was pH 4.0, which exhibited the highest catalytic activity. Under extremely low and high pH, the structure of Fe-loaded MOF-545(Fe) became unstable. Fe-loaded MOF-545 (Fe) may lose its original structure, which decreases the capacity for adsorption and degradation of dyes (Figure S3). The temperature during the reaction was optimized from 10 °C to 60 °C to judge suitable conditions (Figure 3B). The Fe-loaded MOF-545(Fe) exhibited the greatest activity at 20 °C, therefore this temperature was adopted as the common temperature throughout the experiments.

#### 2.2.2. Kinetic Studies of Fe-Loaded MOF-545(Fe) POD-Like Activity

**Figure 4:**
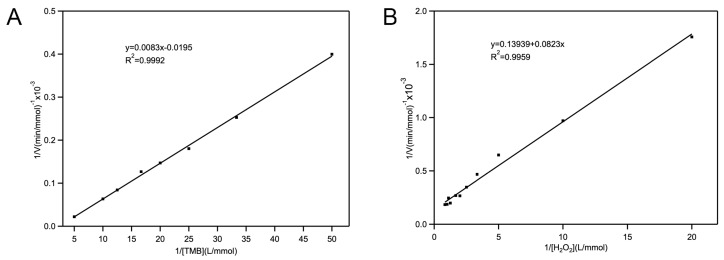
Steady-state kinetic assay of Fe-loaded MOF-545(Fe). The reaction was measured using 1.0 mg·mL^−1^ Fe-loaded MOF-545(Fe) in pH 4.0 buffer at 20 °C. (**A**) The concentration of H_2_O_2_ is 0.5 mM. (**B**) The concentration of TMB was 0.05 mM.

To further investigate the POD-like activity of Fe-loaded MOF-545(Fe), optimization of kinetic studies was performed at pH 4.0 and 20 °C under reaction conditions. The kinetic data were obtained by changing the concentration of one substrate (TMB or H_2_O_2_) while maintaining the others. Typical Michaelis–Menten curves were calculated in the range of the substrate concentration; Figure 4 shows the velocity of this reaction. Both the maximum initial velocity (V_max_) and the Michaelis–Menten constant (K_m_) were calculated from Lineweaver–Burk plots, which are shown in Table 1. The principle of the peroxidase during the catalytic reaction was similar to that which porphyrin formed an enzyme reaction center in H_2_O_2_. In Fe-loaded MOF-545(Fe), the Fe in the center of the heme reacted with H_2_O_2_, which initiated the reaction. Compared with other synthesized peroxidase structures, the Fe-loaded MOF-545(Fe) showed better POD-like activity (Table 1). After reacting with TMB, the structure of the Fe-loaded MOF-545(Fe) was detected using XRD measurement (Figure S4). The results showed that after reacting with TMB, the main structure of Fe-loaded MOF-545(Fe) almost maintained its original structure. The controlled experiments of catalytic activity are shown in Table S1. The Zr-based MOF-545 did not present the catalytic activity. The Fe-loaded MOF-545(Fe) exhibited the highest POD-like activity compared with Fe-TCPP and Fe^3+^. The results showed that loading of Fe ion into the MOF-545(Fe) boosted its POD-like activity. 

### 2.3. The Adsorption of Acid/Basic Dyes

**Figure 5:**
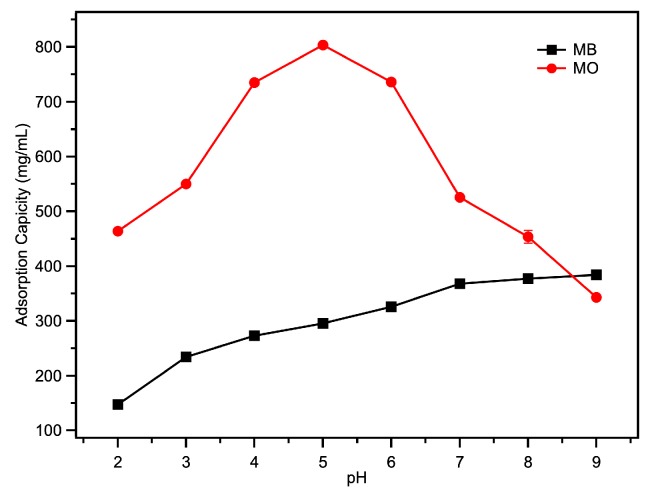
Effect of initial pH on adsorption capacities of Fe-loaded MOF-545(Fe) for MB and MO.

One of the methods through which MOFs are able to remove dyes from aqueous solution is adsorption. Although the Fe-loaded MOF-545(Fe) had the highest enzyme-like activity at pH 4.0, it could still act as a stable absorbent in basic and acidic dyes. MB and MO were used to observe the adsorption of Fe-loaded MOF-545(Fe) as acidic and basic dyes, respectively. The adsorption capacity of Fe-loaded MOF-545(Fe) was observed over a pH range of 2.0 to 9.0, with an initial dye concentration of 10 mg·L^−1^ (Figure 5). MO had a maximum adsorption at pH 5.0 (Figure 5), where the MO was in an acidic form and the Fe-loaded MOF-545(Fe) possessed a positive charge. The results for adsorption with MB and MO were lower and higher, respectively, than the Zr-Based MOF-545 without Fe (Table 2) [24]. The different adsorption abilities were due to the increase of the positive charge on Fe in Fe-loaded MOF-545(Fe). The equilibrium calculations for the adsorption amounts and capacities of MO and MB are shown in Supporting Information (SI) 2.1. 

**Figure 6:**
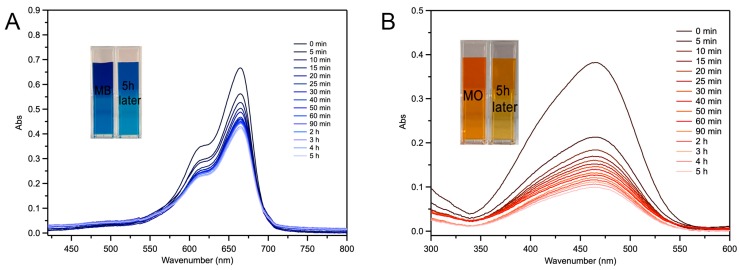
UV-Vis absorption spectra of (**A**) MB, (**B**) MO. Each sample was diluted 2.5 times before UV-Vis absorption spectra. The graph shows the color change before and 5 h after adsorption.

In order to understand the details of adsorption regarding Fe-loaded MOF-545(Fe), UV-Vis absorption spectra were used to detect the spectra changes of MO and MB over time at 464 nm and 664 nm, respectively (Figure 6). The spectra results showed that after 5 min, the intensities of the spectra decreased rapidly for both MO and MB. After about 5 h, the absorbance of MO and MB had decreased by 73.7% and 38.23%, respectively. The absorption spectra results were synchronized with the pH dependence, showing that the adsorption ability of MO in Fe-loaded MOF-545(Fe) was better than MB. The reparative rate of adsorption for Fe-loaded MOF-545(Fe) with MB and MO was over 85% after 8 repeats (Figure S5). The repeatability of Fe-loaded MOF-545(Fe) was much better than most published instances of MOF with a stable structure, which was detected by XRD (Figure S6). These results indicated that Fe-loaded MOF-545(Fe) was appropriately reusable. The stability and repeatability of Fe-loaded MOF-545(Fe) provide advantages to remove dyes from wastewater.

### 2.4. Fe-Loaded MOF-545(Fe) Degradation of the Acid/Basic Dyes

**Figure 7:**
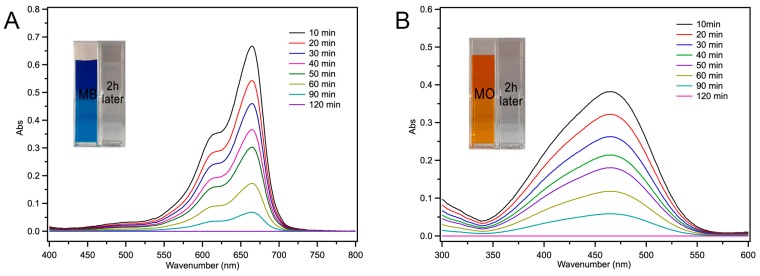
UV-Vis absorption spectra of Fe-loaded MOF-545(Fe) degrading MB (**A**), MO (**B**) with H_2_O_2_. UV-Vis absorption spectra of Fe-loaded MOF-545(Fe) degrading MB (**A**), MO (**B**); each sample was diluted 2.5 times before UV-Vis absorption spectra measurements. The graph shows the color change before and 2 h after degradation process.

In previous work, we already showed that the Fe-loaded MOF-545(Fe) exhibited peroxidase-like, as discussed in Section 2.3. In the early of 1980s, researchers developed the idea of using oxidoreductases like peroxidases to remove dye from wastewater. Peroxidases contain a heme and iron and share the same mechanism. The heme groups of peroxidases are responsible for catalyzing reactions in the presence of hydrogen peroxide [25,42]. The H_2_O_2_ oxidized the heme from its resting state to two powerful oxidants via several steps [43,44]. Therefore, the Fe-loaded MOF-545(Fe) was considered to degrade the dyes with its peroxidase-like activity. Changes were observed over time in the UV-Vis absorption spectra of Fe-loaded MOF-545(Fe), which were reacted with MB and MO with a dye concentration of 1000 mg·L^−1^ at pH 4.0, as shown in Figure 7. After two hours, the dyes were almost degraded completely and changed to colorless (Figure 7 inset). The spectra showing the MO and MB degradation were different from the Zr-based MOF-545, which only adsorbed the dyes [24]. Compared to Fe-loaded MOF-545(Fe) without H_2_O_2_ (Figure 6), the peroxidase activity gave an advantage to remove the dye from wastewater in a short amount of time. The Fe-loaded MOF-545(Fe) not only exhibited absorption capabilities, but also degraded the dyes via a chemical reaction [25]. After 48 h (Figure S7), the spectra were the same as those taken after two hours (Figure 7), meaning that the MB and MO were already degraded completely after a short amount of time (two hours). The MB and MO absorption spectra were not observed in the washing buffer of the depredated Fe-loaded MOF-545(Fe). The results prove our hypothesis in that, when H_2_O_2_ was present, the Fe-loaded MOF-545(Fe) reacted with the dyes as a peroxidase completely and no dyes were absorbed into/onto the Fe-loaded MOF-545(Fe). The structure of Fe-loaded MOF-545(Fe) after 8 cycles of degradation was stable, as shown by the XRD experiment (Figure S8). The adsorption results also showed that Fe-load MOF-545(Fe) exhibited the stable structure after 8 repeats. 

**Figure 8:**
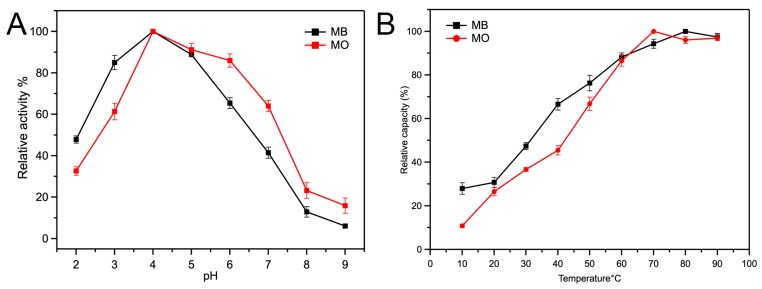

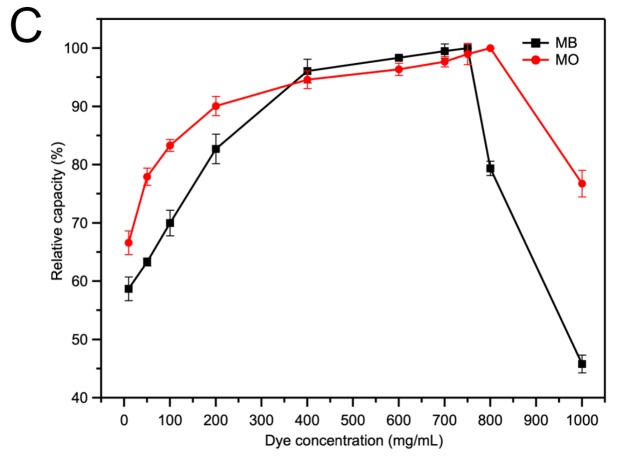
The activity of degradation in MO and MB by Fe-loaded MOF-545(Fe) with H_2_O_2_. (**A**) Degradation of MO and MB from pH 2.0–9.0. (**B**) Degradation of MO and MB from 10 °C to 90 °C. (**C**) Dyes’ concentrations on the activity of degradation MB and MO of Fe-loaded MOF-545(Fe).

To understand the pH affinity for the activity of Fe-loaded MOF-545(Fe), the degradation capacity of Zr-based MOF-545 was detected over a pH range of 2.0 to 9.0 (Figure 8). The degradation capacity gradually increased until 4.0 with increasing pH, but after pH 4.0, the degradation capacity gradually decreased in MB and MO. At pH 4.0, the Fe-loaded MOF-545(Fe) exhibited the highest degradation capacity, which corresponded to the TMB. These results showed that the peroxidase activity of Fe-loaded MOF-545(Fe) was not related to the substrate during the catalytic reaction. When the temperature increased from 10 °C to 90 °C, the degradation capacity of Fe-loaded MOF-545(Fe) increased similarly to most other enzymes. Although the degradation capacity of Fe-loaded MOF-545(Fe) was not affected by the substrate, the concentration of the substrate was limited to the enzyme activity (Figure 8). The highest degradation of MB and MO was observed when the concentrations were at 750 mg·L^−1^ and 800 mg·L^−1^, respectively. When the concentration was greater than 750 mg·L^−1^ in MB and 800 mg·L^−1^ in MO, the degradation rate drastically decreased due to substrate inhibition. Under the same conditions, the degradation capacity of MO was higher than MB, thereby showing that the peroxidase activity of Fe-loaded MOF-545(Fe) was better. 

## 3. Materials and Methods

### 3.1. Materials

Zirconyl chloride octahydrate (ZrOCl_2_·8H_2_O), hydrogen peroxide (H_2_O_2_), and FeCl_3_ were obtained from Shanghai Aladdin Biochemical Technology Co. Ltd. (Shanghai, China). Tetrakis (4-carboxypheneyl) porphyrin (TCPP) was ordered from Tokyo Chemical Industry (Tokyo, Japan). The 3,3′,5,5′-tetramethylbenzidine (TMB) was purchased from Sigma. *N*,*N*-dimethylformamide (DMF) and methanol was purchased from Sinopharm Chemical Reagent Co., Ltd. (Beijing, China). Acetone, formic acid (98%), hydrochloric acid (HCl), and sodium hydroxide (NaOH) were purchased from Beijing Chemical Works (Beijing, China).

### 3.2. Preparation of Fe-Loaded MOF-545(Fe)

ZrOCl_2_·8H_2_O (37.5 mg, 0.111 mmol) was dissolved in DMF (10 mL) into 20 mL disposable scintillation vials and sonicated for 30 min. TCPP (6.5 mg, 0.037 mmol) and formic acid (5 mL) were added to the solution [22,23]. The solution was heated at 130 °C for 72 h. The precipitates were collected and washed with DMF 10 times. Iron chloride FeCl_3_ (100 mg, 0.62 mmol) and Fe-loaded MOF-545(Fe) (50 mg) were dissolved in DMF (10 mL). The solution was heated at 110 °C for 18 h [22]. The microcrystal precipitates were collected and washed with DMF 10 times and then replaced by washing with acetone 10 times. Finally, the acetone was evaporated by heating at 120 °C under a vacuum (30 mTorr) for 5 h. 

### 3.3. Characterization of Fe-Loaded MOF-545(Fe)

The phase structure of the as-synthesized products was identified using a scanning electron microscope (JEOL JEM-6700F) operating at a voltage of 5 kV. X-ray diffraction (XRD, Empyrean) was carried out using Cu–Ka radiation (α = 1.5418 A°) over a 2θ range of 1–25° at a rate of 1°/min with Empyrean (PANalytical B.V., Netherlands). UV-Vis absorption spectra (UV-2700 Shimadzu, Japan) measurements of each MOF were quantitatively metalized. FTIR experiments were measured using an FTIR instrument (Bruker, Germany) with a scan wavenumber range from 2000 to 400 cm^−1^, overlapping 30 times. The surface and porous properties were performed using the ASAP 2020 Accelerated Surface Area and Porosimetry System from Micromeritics (Norcross, GA, USA) at 77 K. The MOF was added to the DMF solution containing 2 M hydrochloric acid at 120 °C for 2 h to remove residual inorganic salt ions and organic molecules before testing [22,45]. 

### 3.4. POD-Like Activity of Fe-Loaded MOF-545(Fe)

The peroxidase-like catalytic activity of Fe-loaded MOF-545(Fe) was determined by the catalytic oxidation of the peroxidase substrate TMB in the presence of H_2_O_2_ by measuring the formation of a blue charge-transfer complex of diamine from TMB at 652 nm (ε = 39000 M^−1^ cm^−1^) [46,47]. The catalytic activity experiment, unless otherwise specified, was conducted in acetate buffer (pH 4.0) in the presence of Fe-loaded MOF-545(Fe) (1 mg·L^−1^) with 0.05 mM of TMB and 0.5 mM of H_2_O_2_. The reaction proceeded at 20 °C with time for 5 min. 

#### 3.4.1. Influence of Reaction pH

The activity of the Fe-loaded MOF-545(Fe) at different pH values was tested using acetate buffer solutions with pH values ranging from 2.0 to 9.0. The reaction was carried out with 1 mg·mL^−1^ of Fe-loaded MOF-545(Fe), to which TMB (0.05 mM) and H_2_O_2_ (0.5 mM) were added. The pH values of the different buffers were adjusted using a pH meter [48]. 

#### 3.4.2. Influence of Incubation Temperature

The catalytic reactions were incubated in different temperature metal baths from 10 °C to 60 °C, under conditions identical to those used for the activity assay [48]. 

#### 3.4.3. Steady-State Kinetics Parameters Analysis

The steady-state kinetics were evaluated by varying one of the concentrations of Fe-loaded MOF-545(Fe), H_2_O_2_ (0–1.2 mM), or TMB (0–0.2 mM) at a time. The reaction was carried out in acetate buffer (pH 4.0) for 5 min and monitored by measuring the absorbency at 652 nm. The kinetic curves were adjusted according to the Michaelis–Menten model [47].

### 3.5. Adsorption of Dyes from Water without H_2_O_2_

The effect of pH for Fe-loaded MOF-545(Fe) on absorption for the basic MB dye and acidic MO dye were examined. The concentration of MB and MO was 10 mg·L^−1^ and the pH was controlled from 2.0 to 9.0 by 0.1 M HCl or 0.1 M NaOH. The 10 mg Fe-loaded MOF-545(Fe) powder was added to 1000 mL of solution without H_2_O_2_ and the mixture was stirred with a magnetic stirrer. The mixture was centrifuged at 10,000 rpm for 30 s after adsorption finished. The absorbance of the supernatant was measured for MO and MB at 464 nm and 664 nm, respectively. 

### 3.6. Degradation of Dyes from Water with H_2_O_2_

The experiment showed effect of the pH for Fe-loaded MOF-545(Fe) on the degradation-like process of adsorption. To observe the effect of degradation in MB and MO, at pH 4.0, the concentrations of MB and MO was graduated with 10 mg·L^−1^, 50 mg·L^−1^, 100 mg·L^−1^, 200 mg·L^−1^, 400 mg·L^−1^, 600 mg·L^−1^, 700 mg·L^−1^, 750 mg·L^−1^, 800 mg·L^−1^, and 1000 mg·L^−1^ with 1.0 mM H_2_O_2_ respectively.

## 4. Conclusions

In this study, Fe-loaded MOF-545(Fe) was observed and characterized via structural and peroxidase-like activity analysis. The POD-like activity of Fe-loaded MOF-545(Fe) was observed by TMB alongside H_2_O_2_. Two representative dyes (basic MB and acidic MO) were used to understand the catalytic mechanism of Fe-loaded MOF-545(Fe). In the presence of H_2_O_2_, the degradation capacity of Fe-loaded MOF-545(Fe) upon reaction with acidic/basic dyes was detected by UV-Vis spectroscopy. More significantly, the results showed that the Fe-loaded MOF-545(Fe) catalyzed the dyes similarly to peroxidase in a short amount of time without any dye being absorbed into/onto it. According to the suitable condition of Fe-loaded MOF-545(Fe) with a stabile structure at varying pH levels over 70 °C, Fe-loaded MOF-545(Fe) also showed good reusability, which is crucial for use. Furthermore, our study demonstrated the advantages for using Fe-loaded MOF-545(Fe) to remove dyes from wastewater.

## Figures and Tables

**Table 1:** Kinetic parameters for different POD-like activity.

	Km (mM)		Vmax (10^−8^M·S^−1^)		Reference
	TMB	H_2_O_2_	TMB	H_2_O_2_	
HRP	0.434	3.70	10.0	8.71	[37]
SiW12@Co_3_O_4_	0.023	167.8	5.3	167.8	[38]
Nickel metal-organic framework	0.365	2.49	6.53	8.98	[39]
Au NPs/PVP–GNs	2.63	104	13.04	11.98	[40]
AuNPs@g-C3N4	0.097	12.3	1.52	9.0	[41]
Fe-loaded MOF-545(Fe)	2.34	1.69	85.4	12	This work

**Table 2:** Comparison of absorption capacity of MB and MO by Fe-loaded MOF-545(Fe) and reported adsorbents.

Adsorbents	Adsorption Capacity (mg g^−1^)		Reference
	MB	MO	
Fe-loaded MOF-545(Fe)	382.35	803.664	This work
PCN-222(MOF-545)	906	589	[24]

## Supplementary Materials

The following are available online at https://www.mdpi.com/1420-3049/25/1/168/s1. Figure S1: FTIR spectra of Zr-based MOF-545 (red) and Fe-loaded MOF-545(Fe). Figure S2: Testing the peroxidase-like activity of MOF-545(Fe). Figure S3: X-ray diffraction (XRD) patterns of Fe-loaded MOF-545(Fe) reaction at different pH. Figure S4: X-ray diffraction (XRD) patterns of Fe-loaded MOF-545(Fe) degrade TMB. Figure S5: Repeatability test of Fe-loaded MOF-545(Fe) for removal of MB and MO. Figure S6: X-ray diffraction (XRD) patterns of Fe-loaded MOF-545(Fe) for removal of MB and MO reused eight times with adsorption. Figure S7: The experiment of Fe-loaded MOF-545(Fe) removed MB(A) and MO(B) by degradation. Figure S8: X-ray diffraction (XRD) patterns of Fe-loaded MOF-545(Fe) for removal of MB and MO reused eight times by degradation. Table S1: Compare the peroxidase-like activity of Fe^3+^, TCPP-Fe and Zr-based MOF-545.
