# Supplementary material for: Fe-Loaded MOF-545(Fe): Peroxidase-Like Activity for Dye Degradation Dyes and High Adsorption for the Removal of Dyes from Wastewater
Source: Molecules. 2019 Dec 31;25(1):168. doi: 10.3390/molecules25010168 (PMC6983047; doi:10.3390/molecules25010168)
Supplement: Supplementary file 1 [file molecules-25-00168-s001.pdf]

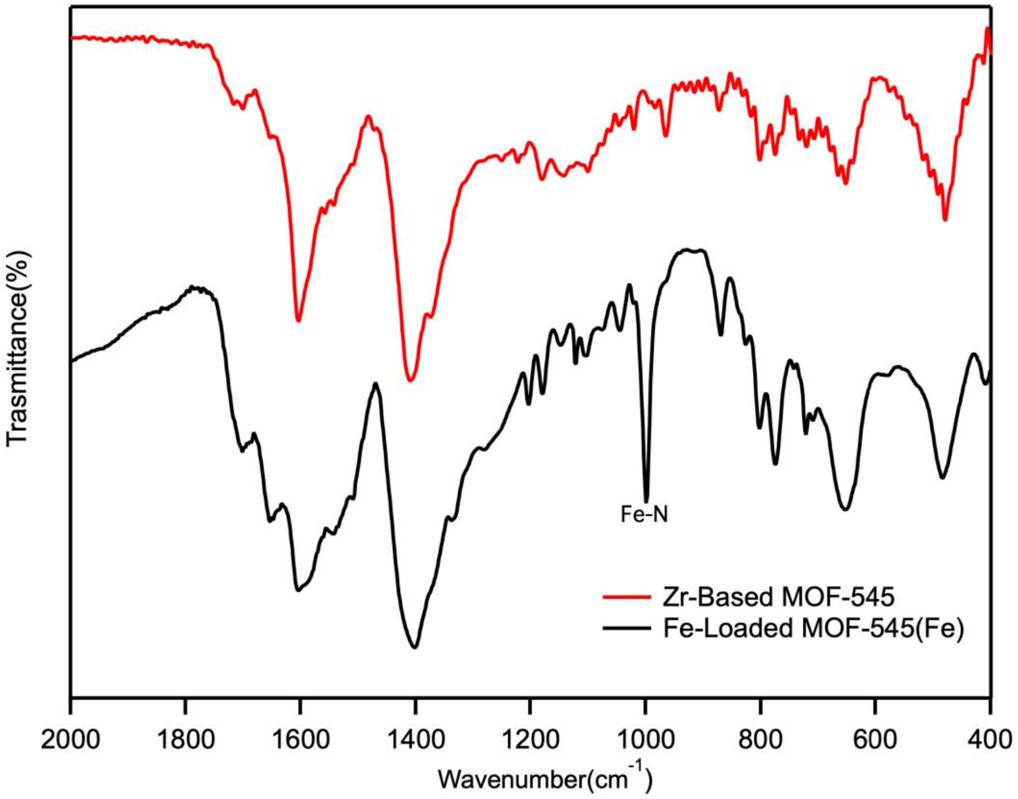

Figure S1. FT-IR spectra of Zr-Based MOF-545 (red) and Fe-Loaded MOF-545(Fe) (black line)

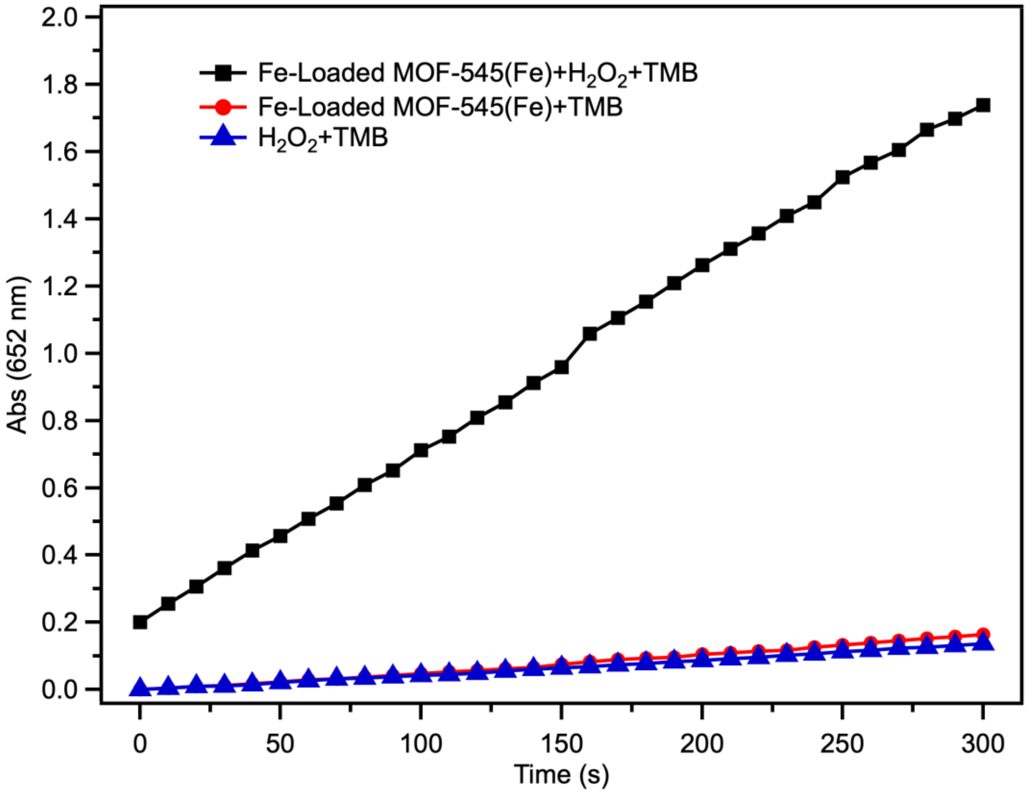

Figure S2. Testing the peroxidase-like activity of Fe-Loaded MOF-545(Fe)

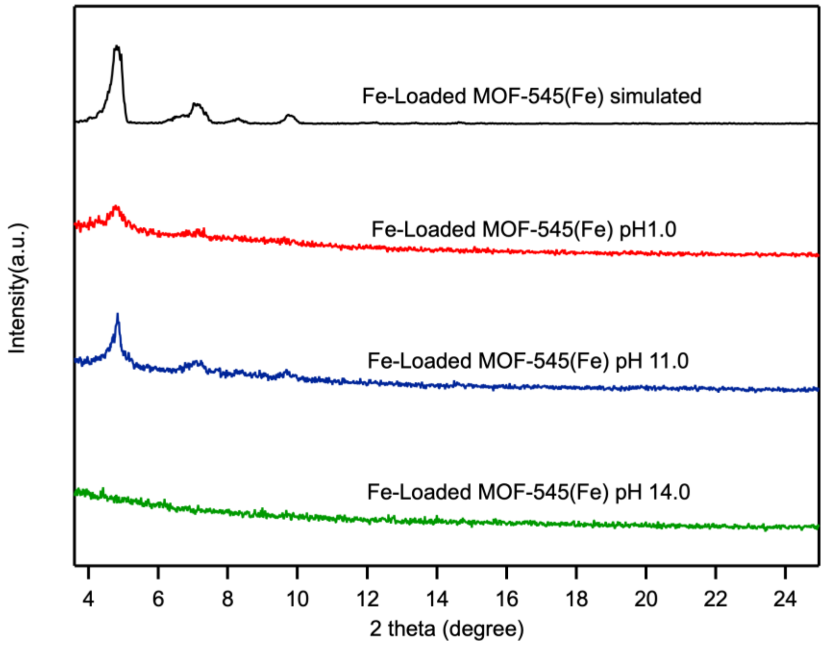

Figure S3. X-ray diffraction (XRD) patterns of Fe-Loaded MOF-545(Fe) reaction at different pH. The simulated Fe-Loaded MOF-545(Fe) (black line), Fe-Loaded MOF-545 (Fe) at pH 1.0 (red), pH 11.0 (blue) and pH 14.0 (green), respectively.

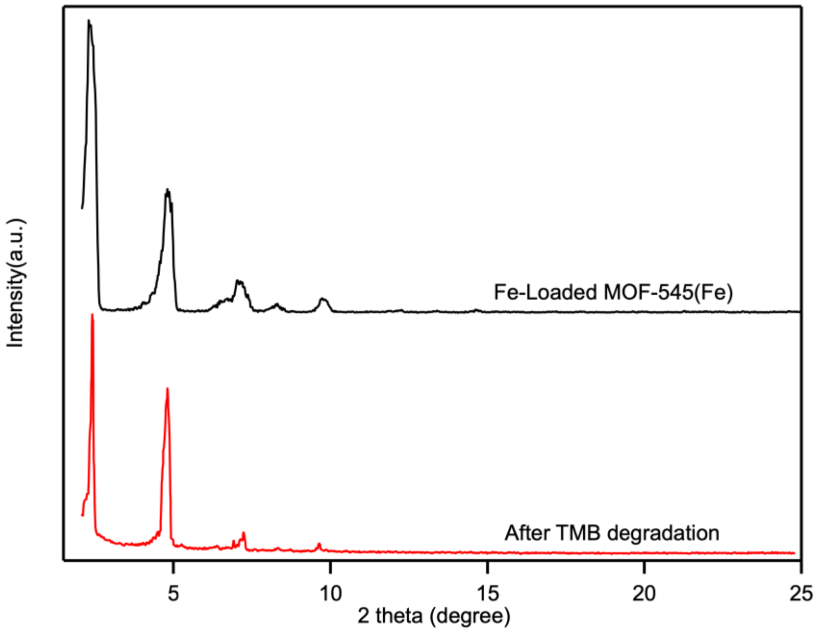

Figure S4. X-ray diffraction (XRD) patterns of Fe-Loaded MOF-545(Fe) degrade TMB. The simulated Fe-Loaded MOF-545(Fe) (black line), the pattern of after TMB degradation (red).

Table S1 Compare the peroxidase-like activity of Fe^{3+}, TCPP-Fe and Zr-based MOF-545

	Fe^{3+}	TCPP-Fe		Zr-based MOF-545		Fe-Loaded MOF-545(Fe)
H_2O_2	+	-	+	-	+	+
TMB	8.29%	0	6.85%	0	0	11.03%

2.1 *The equilibrium calculation for adsorption amount and adsorption capacity of MO and MB*

Define initial time, any time t, equilibrium absorbance value is A_0, A_t, A_e, respectively. Adsorption amount and adsorption capacity was obtained by the following equation:

$$C_t = \frac{A_t}{A_0} \times C_0 \tag{1}$$

$$C_e = \frac{A_e}{A_0} \times C_0 \tag{2}$$

$$q_t = \frac{C_0 - C_t}{m} \times V \tag{3}$$

$$q_e = \frac{C_0 - C_e}{m} \times V \tag{4}$$

In Equation (1)(2), C_0, C_t and C_e(mg·L^{-1}) were represent the dyes concentrations at initial time, any time t and equilibrium time in the solution, respectively. In Equation (3)(4), q_t (mg·g^{-1}) is measured adsorption amount of MOF-545(Fe) at time t, q_e (mg·g^{-1}) was adsorption capacity when adsorption equilibrium is reached. V(L) was the volume of the dye solution and m(g) was the mass of MOF-545(Fe). C_0 was determined prior to the adsorption by measuring the amount of added dye.

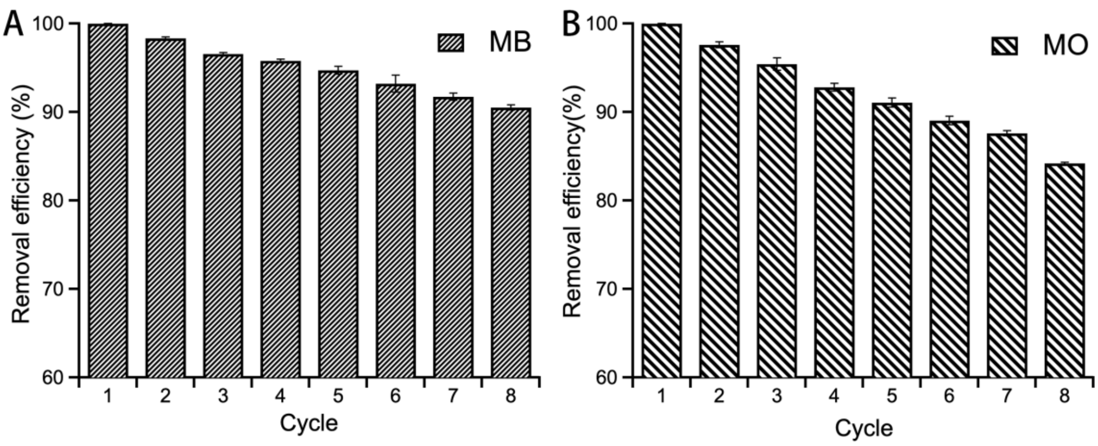

Figure S5. Repeatability test of Fe-Loaded MOF-545(Fe) for removal of MB and MO. The maximum capacity of MB (A) and MO (B) is defined as 100% of the first cycle.

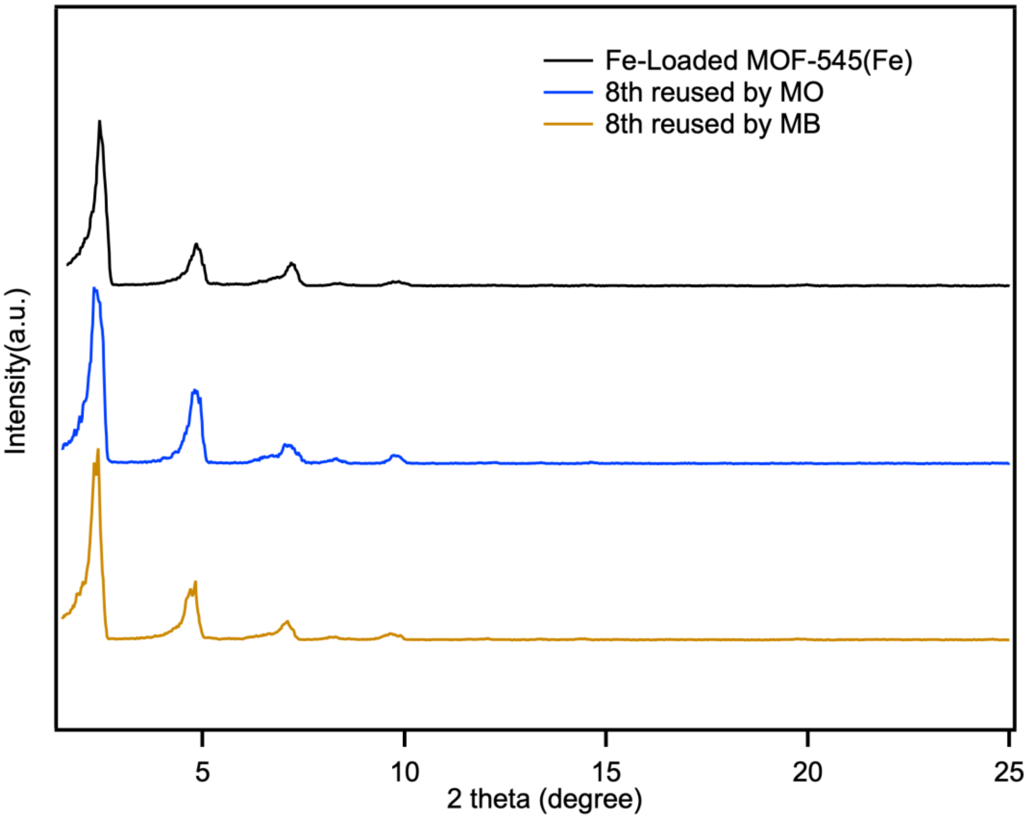

Figure S6. X-ray diffraction (XRD) patterns of Fe-Loaded MOF-545(Fe) for removal of MB and MO reused 8 times with adsorption. The standard Fe-Loaded MOF-545(Fe) (black line), remove MO 8 times of Fe-Loaded MOF-545(Fe) (blue) and remove MB (yellow) 8 times of Fe-Loaded MOF-545(Fe).

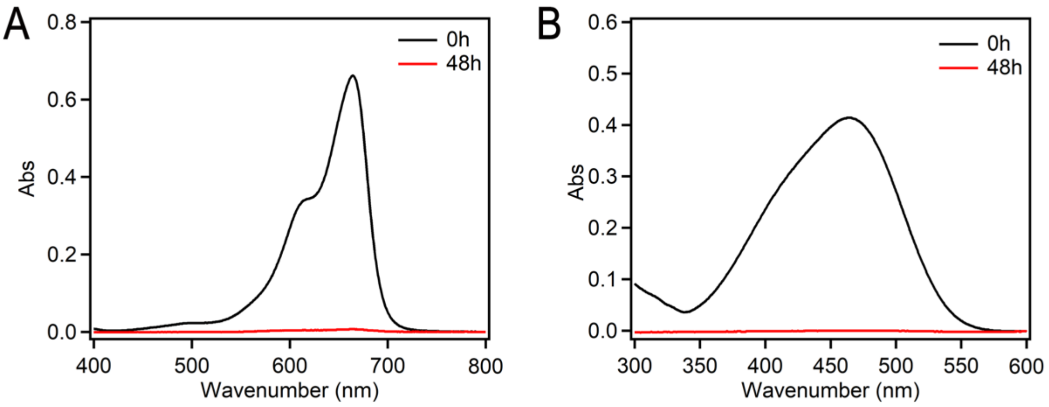

Figure S7. The experiment of Fe-Loaded MOF-545(Fe) remove MB**(A)** and MO**(B)** by degradation. **(A)** The spectra of MB for degradation 48h. **(B)** The spectra of MO for degradation 48h.

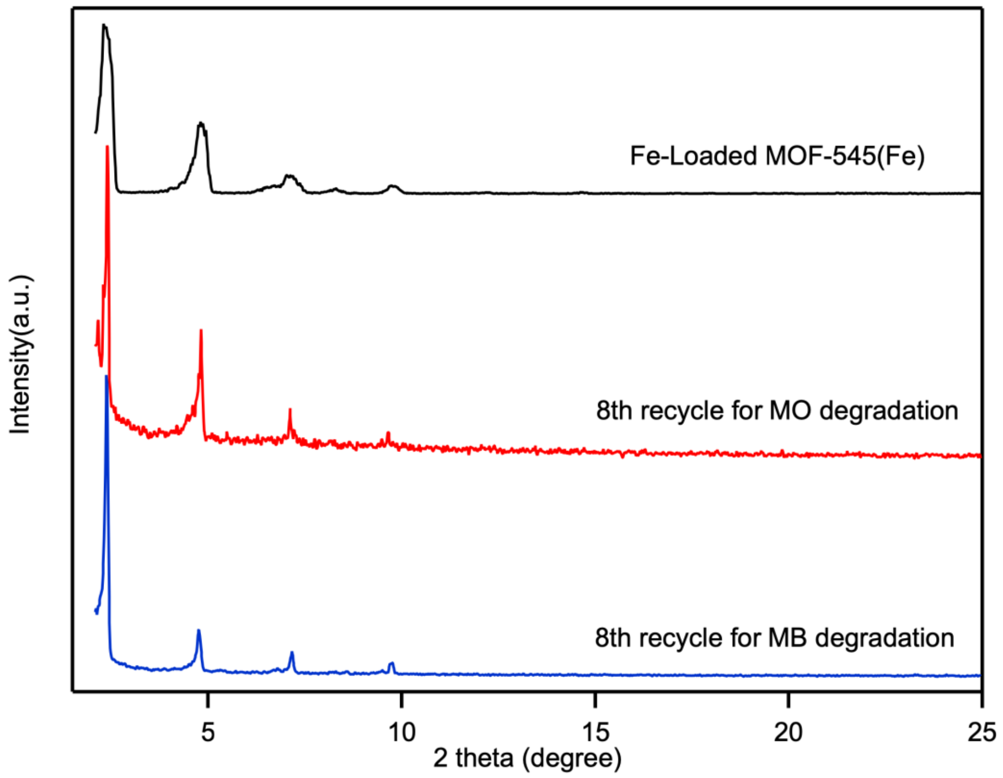

Figure S8. X-ray diffraction (XRD) patterns of Fe-Loaded MOF-545(Fe) for removal of MB and MO reused 8 times by degradation. The simulated Fe-Loaded MOF-545(Fe) (black line), remove MO 8 times of Fe-Loaded MOF-545(Fe) (red line) and remove MB (blue line) 8 times of Fe-Loaded MOF-545(Fe).
